# Correlates of HIV and malaria co-infection in Southern India

**DOI:** 10.1186/1475-2875-11-306

**Published:** 2012-09-03

**Authors:** Ajay R Bharti, Shanmugam Saravanan, Vidya Madhavan, Davey M Smith, Jabin Sharma, Pachamuthu Balakrishnan, Scott L Letendre, Nagalingeswaran Kumarasamy

**Affiliations:** 1 University of California San Diego, San Diego, CA, USA; 2 YRGCARE Medical Centre, Chennai, India; 3 Veterans Affairs Medical Center, San Diego, CA, USA

**Keywords:** Plasmodium vivax, Plasmodium falciparum, Malaria, HIV, Co-infection, Malaria antibody, Retrospective test

## Abstract

**Background:**

Malaria and HIV co-infection adversely impact the outcome of both diseases and previous studies have mostly focused on falciparum malaria. *Plasmodium vivax* contributes to almost half of the malaria cases in India, but the disease burden of HIV and *P. vivax* co-infection is unclear.

**Methods:**

HIV-infected subjects (n=460) were randomly selected from the 4,611 individuals seen at a Voluntary Counseling and Testing Center in Chennai, India between Jan 2 to Dec 31 2008. Malaria testing was performed on stored plasma samples by nested PCR using both genus-specific and species-specific primers and immunochromatography-based rapid diagnostic test for detecting antibodies against *Plasmodium falciparum* and *P. vivax*.

**Results:**

Recent malaria co-infection, defined by the presence of antibodies, was detected in 9.8% (45/460) participants. *Plasmodium vivax* accounted for majority of the infections (60%) followed by *P. falciparum* (27%) and mixed infections (13%). Individuals with HIV and malaria co-infection were more likely to be men (p=0.01). Between those with and without malaria, there was no difference in age (*p*=0.14), CD4+ T-cell counts (*p*=0.19) or proportion CD4+ T-cell below 200/mL (*p*=0.51).

**Conclusions:**

Retrospective testing of stored plasma samples for malaria antibodies can facilitate identification of populations with high rates of co-infection, and in this southern India HIV-infected cohort there was a considerable burden of malaria co-infection, predominantly due to *P. vivax*. However, the rate of *P. falciparum* infection was more than 6-fold higher among HIV-infected individuals than what would be expected in the general population in the region. Interestingly, individuals co-infected with malaria and HIV were not more likely to be immunosuppressed than individuals with HIV infection alone.

## Background

HIV, a sexually or parenterally transmitted viral infection, and malaria, a mosquito-borne parasitic disease, are two disparate and deadly diseases that are often brought together by overlapping geographic distributions. When HIV and malaria co-infection occur in the same individual, both diseases are adversely impacted. HIV-infected individuals are at increased risk of: acquiring malaria [1] developing severe malarial disease [2,3], recrudescing malaria symptoms [4], and experiencing treatment failure of malaria [5]. Additionally, acute malaria is associated with an increase in HIV viral load [6] and a steeper decline in CD4 cell count [7], and these viral load and CD4 count changes can take several weeks to recover after successful malaria therapy [6]. These interactions may facilitate spread of both diseases [8]. These observations have largely been made in the setting of HIV co-infection with *Plasmodium falciparum* in sub-Saharan Africa. Much less is known whether *Plasmodium vivax* has similar interactions with HIV. Therefore, this study was conducted to: i) determine the prevalence and risk factors of malaria co-infection in a cohort of HIV-infected individuals in southern India, a region with predominantly *P. vivax* malaria and ii) evaluate the strategy of using stored specimens for quick retrospective assessment of populations for co-infection burden.

## Methods

### Study population

The subjects were randomly selected (10%) from the 4,611 HIV-1 positive individuals seen at the Voluntary Counseling and Testing center of Y. R. Gaitonde Center for AIDS Research and Education (YRGCARE) between Jan 2, 2008 and December 31, 2008. They were all newly diagnosed with HIV-1 infection and were not receiving antiretroviral therapy. The study was approved by the ethics boards of the University of California San Diego, YRGCARE, and the Indian Council of Medical Research. All volunteers provided written informed consent. Blood samples were processed immediately following collection and plasma stored in −70°C freezer for a period ranging from 18 to 24 months before being evaluated in the current study.

### HIV and CD4+ T-cell count

Blood (6 mL) was collected in EDTA tubes (catalog no. 367861, BD, USA) and plasma separated after centrifugation at 2,500 RPM for 12 min. Tests for diagnosing HIV were either Determine HIV 1/2 test (Abbott Laboratories), Signal HIV Rapid Test (Span Diagnostics Ltd., India), or First Response HIV 1–2.0 (PMC Medical Pvt. Ltd., India). CD4+ T-cell counts were determined by flow cytometric panLeukogating method (Beckman Coulter, USA).

### Malaria PCR

DNA was extracted from 200 μL of plasma sample using QIAamp DNA Blood Mini Kit (catalog no. 51106, Qiagen, USA). Nested PCR was done using both genus-specific and species-specific primers (Table 1) targeting the *Plasmodium* spp. 18S small subunit ribosomal RNA genes [9,10]. The first PCR was performed in a total volume of 20 μL containing 3 μL of extracted DNA, 17 μL of i-Master Mix PCR Kit (catalog no. 25201, Intron Biotechnology, Korea), and forward and reverse primers (0.2 μM). The nested species specific PCR was performed in a total volume of 20 μL containing 1 μL of PCR product. DNA, extracted from plasma of microscopy confirmed malaria positive (*P. falciparum* and *P. vivax*) and malaria negative individuals, was used as positive and negative control, respectively. To confirm the presence of amplifiable human DNA, 20% (90/460) samples were randomly selected and underwent PCR using primers for the human rRNA gene p53 [11]. 

**Table 1:** **Primers for nested PCR of *Plasmodium *18S rRNA gene **

**Species**	**Primer**	**Sequence**	**Size of PCR product (bp)**
*Plasmodium sp.*	rPLU5	CCTGTTGTTGCCTTAAACTTC	1,200
	rPLU6	TTAAAATTGTTGCAGTTAAAACG	
*P. falciparum*	rFAL1	TTAAACTGGTTTGGGAAAACCAAATATATT	205
	rFAL2	ACACAATGAACTCAATCATGACTACCCGTC	
*P. vivax*	rVIV1	CGCTTCTAGCTTAATCCACATAACTGATAC	120
	rVIV2	ACTTCCAAGCCGAAGCAAAGAAAGTCCTTA	

### Malaria antibody testing

An immunochromatography-based rapid diagnostic test kit, SD BIOLINE Malaria Pf/Pv Kit (Catalog No. 05FK30I-IN-02, SD Bio Standard Diagnostics Pvt. Ltd., India), was used for detecting antibodies (IgG, IgM, IgA) against merozoite surface protein (MSP) of both *P. falciparum* and *P. vivax*. Each of the 460 plasma samples was tested individually for malaria antibodies. Blood smears were not available for examination by microscopy.

### Statistical analyses

Student *t*-test was used for continuous variables and Chi-square test for categorical variables (IBM SPSS Statistics Version 19). Non-parametric test, Mann–Whitney *U* test, was used where appropriate. One-way ANOVA was done to compare characteristics of the 3 malaria positive subgroups.

## Results

Participant ages ranged from 21 to 68 years and 63% were male. Table 2 shows the demographic characteristics of the study cohort. *Plasmodium* antibodies were detected in plasma from 45 of the 460 participants (9.8%). The majority of the infections were due to *P. vivax* (60%) followed by *P. falciparum* (27%) and mixed infections (13%). Three-fourths of the co-infected individuals were in the 30-50 year range, but there was no difference in average age between those with and without malaria (38 vs. 40 years, *p*=0.14), median CD4+ T-cell counts (222 vs. 244/μL, *p*=0.19) or proportion CD4+ T-cell below 200/μL (48% vs. 40%, *p*=0.51). The co-infected subjects, however, were more likely to be male (80% vs. 61%, *p*=0.01). Further analysis of the malaria positive groups (*P. vivax, P. falciparum*, and mixed infections) did not show any significant difference in the above characteristics (age, gender, median CD4+ and proportion below 200/μL). Parasite DNA was not detected by PCR in any of the samples. Positive and negative controls were used with each batch of specimens processed and performed as expected. Amplifiable human DNA, tested using primers for the p53 gene, was found in 93% (84/90) of the randomly selected samples.

**Table 2:** Demographic characteristics of the study cohort

	**Malaria Neg**	**Malaria Pos**	***p*value**
	**(N=415)**	**(N=45)**	
Mean age, y (±SD)	38 ± 9	40 ± 9	0.14
Gender (Number (%) men)	253 (61%)	36 (80%)	0.01
CD4+ cell count			
Median, (IQR),/μL	222 (89, 403)	244 (140, 571)	0.19
Proportion below 200/μL	48% (175/363)	40% (15/38)	0.51
*Plasmodium* species			
*P. vivax*		27 (60%)	
*P. falciparum*		12 (27%)	
Mixed infections		6 (13%)	

## Discussion

In India, the potential for intersection of malaria and HIV epidemics is great but data on co-infections are sparse. Two studies, one conducted in Chennai and the other in Mumbai, have identified co-infected individuals in hospital-based settings [12,13]. However, this study is more representative of the community and is the first one to demonstrate a high *P. vivax* co-infection rate. The majority of the infections in this HIV-infected cohort were due to *P. vivax* (60%), which is similar to that in the general population in the state of Tamil Nadu [14]. In contrast, compared to the general population [15], the co-infected cohort had a lower proportion of *P. vivax* (60% vs. 96%); higher proportion of *P. falciparum* (27% vs. 4%); and a considerable number of mixed infections (13%). The higher prevalence of falciparum malaria in HIV-infected individuals has implications for clinical management. Incorrect identification of *Plasmodium* species can lead to inappropriate malaria treatment because chloroquine is still the first-line treatment for *P. vivax*[16], but would be ineffective for falciparum malaria due to high prevalence of chloroquine resistance [17].

There was a preponderance of men (80%) with a mean age of 40 ± 9 years in the co-infected group, which mirrors the trend seen in the general population [14]. The high prevalence of malaria co-infection in men may be related to occupation and travel that puts them at an increased risk of being bitten by infected mosquitoes and needs to be investigated further. Interestingly, this study did not find an association between lower CD4+ T-cell count and malaria co-infection. This is in contrast to other studies where subclinical and symptomatic falciparum malaria was more common in HIV-infected compared with HIV-uninfected individuals and the risk increased with advancing immunosuppression [1,18,19]. Malaria in this cohort was mainly due to *P. vivax* and it is speculated that immunosuppression due to HIV may not increase the risk of vivax malaria co-infection, but more studies are needed to confirm this finding.

Malaria diagnosis was made by detection of malaria antibodies by the SD BIOLINE kit. Although, its sensitivity is much lower than microscopy or nested PCR and ranges from 47% to 69% [20,21], this method is well suited to detect malaria prevalence in low endemic areas [22,23]. The anti-MSP antibodies measured have very short half-lives (9.8 days) [24] that peak by day 7 and are cleared by day 28 [25]. In the absence of repeated infections in a region with unstable malaria transmission, this suggests that malaria infection in this co-infected group was recent, probably within a range of 1-4 weeks. Although data on symptoms or malaria treatment history was not collected, it is presumed that subjects were asymptomatic as they were seen in a HIV testing center and not a treatment clinic. No parasite DNA was detected using PCR-based methods. There could be several explanations for this. First, parasite DNA in stored plasma samples may have degraded. This is unlikely, as the authors have earlier shown that PCR is able to detect *Plasmodium* DNA in serum samples frozen up to 2½ years [26]. The current samples were more recent (less than 2 years old) and better preserved (stored at −70°C). Another point supporting specimen integrity is the presence of amplifiable human DNA in 93% of selected samples. Second, parasite DNA levels may be below the detection limit of PCR. Although, the sensitivity of the nested PCR method is high and ranges from 0.5-1 parasite/μL [27,28], plasma samples can have PCR yield up to 3-fold lower than whole blood [29]. Another reason for low parasitaemia levels is the high likelihood of subclinical infection among the participants. Real-time PCR may have a higher [30] or similar [21,27] sensitivity compared with nested PCR. Longitudinal data is lacking to confirm whether some individuals went on to develop symptomatic malaria or detectable parasite DNA. Further, tests were done only for *P. falciparum* and *P. vivax*, the two predominant *Plasmodium* species in the region and may have missed infections caused by other spp. like *Plasmodium ovale* and *Plasmodium malariae* although their contribution is unlikely given the antibody results.

## Conclusion

Taken together, although malaria antibody test cannot be used to make a clinical diagnosis, it is a preferred method for retrospective malaria prevalence studies in regions with unstable malaria transmission, as in this study region.

## Competing interests

The authors declare that they have no competing interests.

## Authors' contributions

ARB, DMS, SLL, and NK conceived the study, SS, VM, JS, and PB implemented the study. ARB and DMS undertook data analyses, interpretation of results and drafted the manuscript. SLL and NK were involved in critical revision of the manuscript. All authors read and approved the final manuscript.
